# formr: A study framework allowing for automated feedback generation and complex longitudinal experience-sampling studies using R

**DOI:** 10.3758/s13428-019-01236-y

**Published:** 2019-04-01

**Authors:** Ruben C. Arslan, Matthias P. Walther, Cyril S. Tata

**Affiliations:** 1 Center for Adaptive Rationality, Max Planck Institute for Human Development, Berlin, Germany; 2 Technical Workgroup, University of Münster, Münster, Germany; 3 Technical Workgroup, University of Göttingen, Göttingen, Germany

**Keywords:** Survey, Study, R, Web, Online, Feedback

## Abstract

Open-source software improves the reproducibility of scientific research. Because existing open-source tools often do not offer dedicated support for longitudinal data collection on phones and computers, we built formr, a study framework that enables researchers to conduct both simple surveys and more intricate studies. With automated email and text message reminders that can be sent according to any schedule, longitudinal and experience-sampling studies become easy to implement. By integrating a web-based application programming interface for the statistical programming language R via OpenCPU, formr allows researchers to use a familiar programming language to enable complex features. These can range from adaptive testing, to graphical and interactive feedback, to integration with non-survey data sources such as self-trackers or online social network data. Here we showcase three studies created in formr: a study of couples with dyadic feedback; a longitudinal study over months, which included social networks and peer and partner ratings; and a diary study with daily invitations sent out by text message and email and extensive feedback on intraindividual patterns.

In the wake of the replication crisis in psychology, greater transparency for the scientific process has been advocated as a way to improve reproducibility (Chambers, 2013; Open Science Collaboration, 2015). Researchers should ensure reproducibility not just for the data analysis but also for the process of data collection and the software tools used for it.

Many software packages for online survey data collection are commercial and closed-source. This can be a sustainable solution to the dearth of academic funding for scientific software maintenance. Still, there are drawbacks: Many survey companies do not primarily cater to academics, and therefore do not include features that are desirable mainly for that audience, such as (1) reproducibility, (2) traceability, (3) privacy guarantees, and (4) extensibility. (1) Reproducibility is impoverished because potential replicators have to invest time, money, or both into reproducing the study setup, either because they lack access to the same software or because the software or study authors have made no reproducible study setups available. To make studies reproducible would mean making their structures easily exportable to open formats that can be read by other software packages.^1^ Commercial developers are not incentivized to develop such formats and export capabilities; it is more profitable to lock users in by making it hard for them to switch to a different product and take their data and study setup with them*.* (2) Although commercial software developers do need to fix bugs to keep their users satisfied, they have few incentives for ensuring traceability and accountability, such as by communicating a data recall due to software errors to researchers whose work might be affected (e.g., see Feldman, 2019). Other researchers do not have the means to trace studies with potential errors because researchers usually do not cite the precise version of the software that was used; indeed, versioning may not even be visible to the users. (3) Strong privacy guarantees defy the business models of many data collection apps, which can profit off interlinked data and which can save money by using cloud services. Academic researchers, on the other hand, must be wary of fostering distrust in their users by using software that siphons more data than it needs to.^2^ (4) Finally, psychological researchers often want to include specialized measures—for example, to collect reaction time data. Although some commercial products are extensible, support from providers for third-party extensions is often poor.^3^

Noncommercial open source software for online survey data collection does exist, but many products are no longer in active development.^4^ Others, such as LimeSurvey, offer a free core software that requires technical skill and funds to self-host alongside a commercial-hosted solution. Because colleagues found no existing free software tools that satisfied their needs when seeking to conduct long-term longitudinal studies, diary studies, and experience-sampling studies, we therefore began developing formr in August 2013. The main goal was to build a study framework that would address the needs of the longitudinal research for which it was designed, but also be flexible enough to cater to unanticipated needs. Because the target group included researchers in psychology and other social sciences, not marketers or students, we prioritized flexibility, the potential for automation, and shareability, which is closely linked to reproducibility. Therefore, we consciously decided against implementing a point-and-click survey design interface. We also tried to use existing software libraries whenever possible, to be able to focus on the specific capabilities needed in formr. We have been developing new features and doing maintenance work continuously since 2013, and free hosting has been available since 2014.^5^ The present article does not contain a feature comparison with all major competing tools (such as Qualtrics, LimeSurvey, Questback, and Surveymonkey), because so many exist and it is often very difficult to find out whether several specific study designs can be implemented using a tool without acquiring expertise with the tool. However, we and a group of other researchers have started work on a living document that will maintain such feature comparisons, which might be useful to others and could be collaboratively extended.^6^

## Components

The formr framework features three main components. The first is a simple **survey** framework that allows researchers to pose questions to users and collect information from them. The resulting surveys are comparable to those from many existing solutions. The second component is the study control framework, called **run** in formr for historical reasons. It allows researchers to manage access to a study, organize who answers which questions and when, send invitations or reminders via email or text message, give feedback to users, and so on. The first two components are written in PHP and are tightly integrated. It is not possible to use the surveys without at least a minimal setup of the study control framework, but it is possible to use the control framework without using formr surveys. Researchers can substitute surveys from other sources, such as SoSciSurvey (Leiner, 2014) or LimeSurvey (Limesurvey GmbH, 2012), for ours within the framework, although they will thus sacrifice some of the integrated capabilities. The last component is the utility **R package**. It is independent of the PHP software and makes some common operations, such as setting timeouts or cleaning and aggregating the item data, easier.

Our framework owes its extended capabilities and flexibility to OpenCPU^7^ (Ooms, 2014), which provides a principled way to execute R code securely via a Representational State Transfer application programming interface (RESTful API).^8^ By integrating formr with OpenCPU, researchers familiar with R can use their favorite packages in formr. Deeply integrated packages, including R Markdown (Allaire et al., 2018) and knitr (Xie, 2018), facilitate dynamic response generation. Other tidyverse (Wickham, 2017) packages, such as lubridate (Spinu, Grolemund, & Wickham, 2018) and dplyr (Wickham, François, Henry, & Müller, 2018), help control the study flow, whereas ggplot2 (Wickham, Chang, et al., 2018) and various HTML widgets (Vaidyanathan, Xie, Allaire, Cheng, & Russell, 2018), such as rbokeh (R. Hafen & Continuum Analytics, Inc., 2016), enable graphical feedback to users.

## Security, stability, and the general data protection regulation (GDPR)

The security model of formr.org is simple. By default, no external services get access to any user data, ever. Users can optionally connect formr to email servers and, for instance, text-messaging engines, but they can restrict the user data in such connections to an anonymous user token. All connections to formr.org are forced to be encrypted via HTTPS (also called HTTP over Transport Layer Security). We host our own instance of the OpenCPU software, and its connection to the formr software is also encrypted. No participant-related data are exposed except when researchers export the data or when formr automatically computes feedback and similar data-based operations. In these cases, both data entry and feedback are protected by long cryptographic tokens that function as strong passwords. We encourage and teach researchers to pick strong passwords for the admin area. We store passwords only as salted bcrypt hashes that are robust to brute-force attacks and rainbow tables. Encrypted backups of our database are stored nightly, and additional safeguards against accidental data deletion are in place. Studies set a single, study-specific cookie that can be set to expire after the end of the session, and a GDPR cookie notice is displayed until it is dismissed. Because formr.org is hosted at the University of Göttingen, is not incorporated, and uses state-of-the-art security, we expect it to fulfill reasonable standards of security. However, Health Insurance Portability and Accountability Act (HIPAA) or GDPR compliance have not been certified, as we lack the means to pay for the necessary legal advice. Ultimately, users of formr are responsible for informing participants and getting the appropriate consent. Potential users who are not satisfied by this still have the option to host formr, if they can provide for state-of-the-art security in hosting.

## Survey framework

The survey framework offers the most common basic functionality needed for online data collection. The information to be collected is specified in a spreadsheet, or *item table*. Item tables must contain a column for the item names under which the recorded information will be stored and a column for the item type. Item tables can be uploaded when creating or updating a survey on formr.org. There, item tables are translated to HTML widgets in the web browser, providing users the ability to enter input. More than 30 input types are available. Customizing new item types is possible and extremely flexible, because researchers can load custom JavaScript. This functionality has been used in the past to implement event history calendars as Gantt charts (Wieczorek et al., 2018), to enable drawing, and so on.

The first line of the spreadsheet must contain the column names. Column names that are not part of the specification (e.g., English translations or comments) are ignored. From our experience with collaborative study design, we have found it most convenient to use Google Sheets^9^ to work jointly on item tables, but it is also possible to use local Excel files, OpenDocument spreadsheets, or simply comma-separated value (CSV) or tab-separated value (TSV) files. Google Sheets is the closest thing to a visual survey designer that formr has to offer, since it can be synchronized with formr with just one click. The easiest way to familiarize oneself with the item table capabilities is to start from the demo all_widgets spreadsheet^10^ that we made to illustrate the capabilities of formr.

The standard for item tables is loosely based on the XLSForm standard,^11^ so that simple tables should be transferable between software products using this standard and formr with little effort. We do not completely implement the standard in places where we found less technical terms, thought the control framework accomplished researchers’ needs better, or have implemented functionality that was not part of the standard. Still, interoperability with other implementations of XLSForm should be substantial.

### Item table columns

Apart from the *type* and *name* columns, several others usually can and will be specified.

The *label* column specifies text that will be shown to the user. It can be simple text, or it can be marked up using HTML^12^ or Markdown^13^—a simple, readable way of marking up text that is familiar to many R users and requires minimal effort to learn. Labels can be made dynamic by integrating R code, as in knitr or R Markdown.^14^ This feature can be used to customize labels—for example, to greet a user on the basis of their reported gender, to indicate which object is to be rated, to generate dynamic items for a test, or to give personalized feedback during or after the study.

The *optional* column can be used to designate items as optional. By default, all items require a response (i.e., if respondents have not answered the item, they cannot continue to the next part of the survey), except for items such as sliders and checkboxes, for which “nothing” is a valid answer. The validity of a response is checked before submission to the server, using the HTML5 validation standard^15^ (Hunt, 2010) or the JavaScript-based webshim framework (Farkas, 2016), where necessary. Because client-side checks (such as HTML5 validation and JavaScript) can be circumvented by technologically capable users, the checks are repeated on the server side.

The *showif* column allows researchers to specify that an item will be shown conditionally. If this column is left empty, the item will always be shown. If it contains a condition (e.g., *in_a_relationship == 1*), the item will be shown only if the condition evaluates to TRUE. There are two ways to use showif. First, if an immediate change depending on data on the same page is desired (e.g., showing a text box only if *gender == “other”*), the showif will be evaluated with JavaScript and verified later on the server side using R. Second, if previously collected data are referenced (e.g., the gender of the participant in the diary, not collected on the same page), the showif is evaluated with R and the necessary data are made available automatically. These two modes exist so that formr sends out no entered data unless the data are explicitly requested by the researcher, as a security precaution. The two modes can be combined, but it is rarely necessary to do so, or to even think about them as separate modes.

The *value* column can be used to prefill the values for items—for instance, to verify that previously entered information (e.g., contact data from the previous wave) is still up to date. In this way, the participant will not have to reenter previously given responses, but still can make changes if needed. The column is also used to perform hidden calculations in the survey using R—for instance, with the *calculate* item type.

The *class* column allows researchers to assign a cascading style sheet^16^ (CSS) class to the item in order to style it visually. Many preset classes are available, and researchers can customize their own in the study control framework settings.

**Fig. 1:**
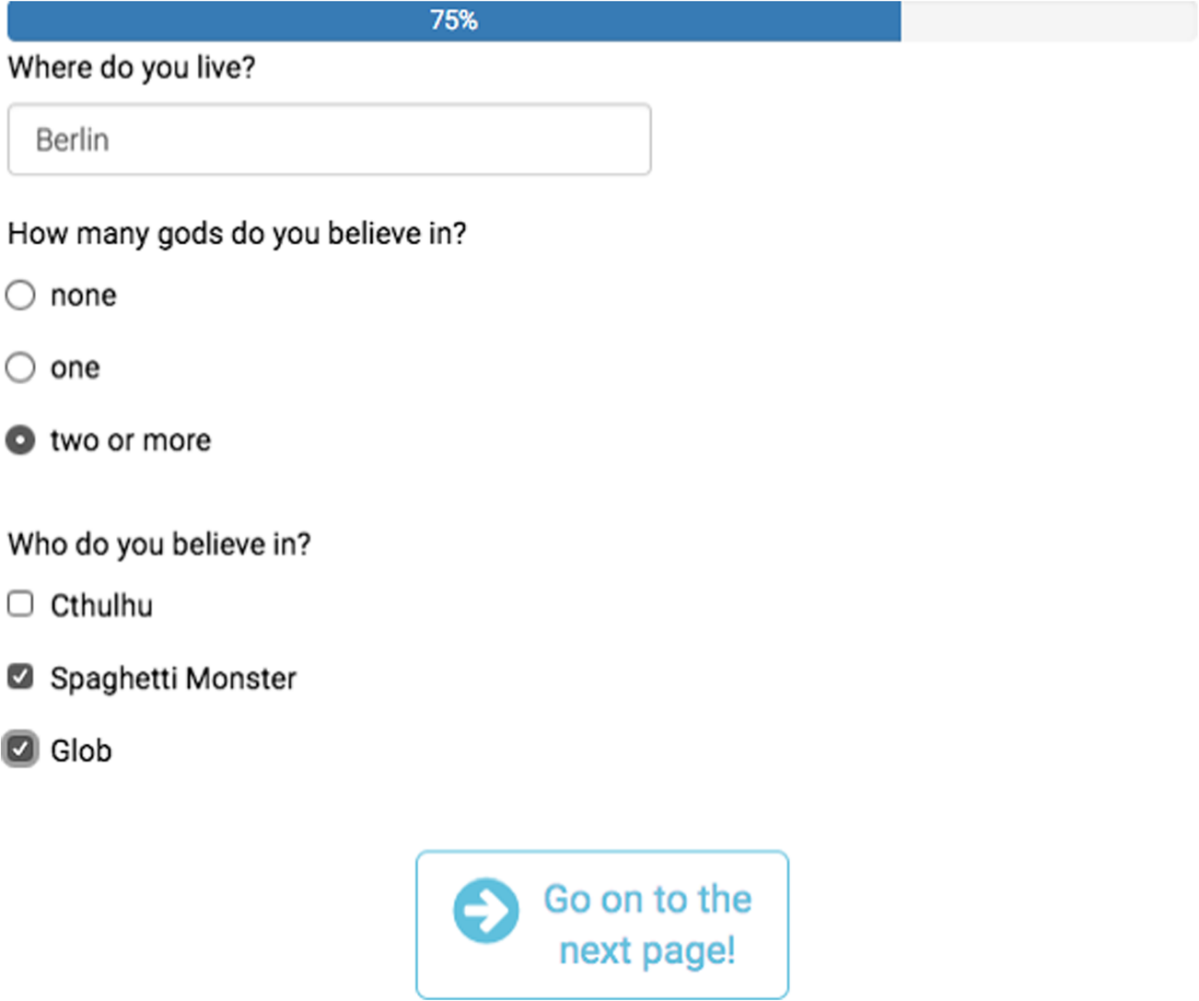
Table 1, as rendered by formr on a mobile device

Finally, the columns *choice1* to *choice12* allow researchers to easily define the choices for multiple-choice items. The number of the choice item is stored as the response to the corresponding survey question, not the label. Some researchers want more flexibility—for instance, they may need more than 12 choices, may want to store text instead of consecutive numbers for the choices, or may wish to reuse a choice list frequently. This flexibility can be achieved by specifying a second sheet in the spreadsheet that list choices row by row with a *name* and a *label* column. The specified *list name* can then be referred to as the second word in the *type* specification (e.g., *mc likert5point*). Tables 1 and 2 show a simple example table (the survey and the choices sheet), and Fig. 1 shows how formr would render this table.

**Table 1:** Simple example item table

type	name	showif	label	choice1	choice2	choice3
text	abode		Where do you live?			
mc god_nr	god_nr		How many gods do you believe in?			
mc	god	god_nr == 1	Who do you believe in?	Cthulhu	Spaghetti Monster	Glob
mc_multiple	gods	god_nr == 2	Who do you believe in?	Cthulhu	Spaghetti Monster	Glob

This survey contains three question types. Conditionally on the response to the second question, the third or fourth questions can be shown

**Table 2:** Simple example choices for the item table shown in Table 1

list_name	name	label
god_nr	0	none
	1	one
	2	two or more

## Results storage

The results for every survey are stored in two tables: a (primary) wide table and a (secondary) long table. In the wide table, the item names provide the columns. Five additional columns—*session*, *created*, *modified*, *ended*, and *expired*—index information about the survey participation. The *session* column is an identifier for the individual and can be used to merge data across surveys. For single surveys, it will be unique in the dataset. The combination of *session* and *created* is unique even in repeated surveys.

The long table is useful mainly if item orders were randomized or to examine response times and patterns on the item level, but it is also “tidy” data in the sense of the tidyverse framework (Wickham, 2017). It can be found under *detailed results*. It has one row per item response, and correspondingly, the columns include *session*, *item_name*, *answer*, *created*, *shown*, *saved*, *display_order*, *hidden*, and *answer*. These fields allow researchers to investigate nonresponse and response times according to the item characteristics (Bosnjak & Tuten, 2001).

Both results tables can be exported directly from formr to CSV, TSV, Excel, and JSON. Using the formr R package, researchers can also easily generate SPSS and Stata files (including item metadata such as labels) with the help of the haven package (Wickham & Miller, 2018). Research and personal data can be deleted by researchers in aggregate or on a participant level, when they are no longer needed or when requested. After warning researchers, we plan to expunge personal data and backups after legally mandated time frames, as well.

## Data privacy measures

At the survey level, researchers can set surveys to be *unlinked* or to *hide results*. Unlinking a survey means that the results will only be shown in random order, without user codes or dates, and only after a minimum of ten results are in. Researchers can use this feature to disconnect surveys containing personally identifiable information from surveys containing research data. Email contact and payment are then possible by automation in formr. As a further step, researchers can also disable the display of results completely. Once turned on, these features cannot be turned off again. In this way, researchers can allow research assistants to manage a study in formr without having to trust them to maintain participants’ privacy.

## Referring to past results

In any place where R can be used in formr, formr automatically parses the code for survey and variable names. So, for the showif *god_nr == 1*, formr will determine that it refers to the variable *god_nr* in the same survey session and automatically make the data for this variable available in R. In the showif *screening$age > 21*, formr will first notice the reference to the survey name *screening*, if it is part of the same study, and then the reference to the variable therein. The previous entries by this participant in the screening survey will then be made available as a data frame in R. Generally, this means that researchers can refer to data stored anywhere in the study and expect it to be automatically available without further ado, in a format very similar to how they would expect it in a data analysis. In some cases, when variables are not explicit in the code, it is necessary to list them explicitly so that formr will make them available. Because having data available automatically is so convenient, it is very important to link related surveys in the study framework.

## Study framework (runs)

Our aim for the study framework (called *runs* in formr, for historical reasons) was to offer high flexibility while still making common study designs easy to implement. To this end, we implemented a programming environment using IF conditions and GOTO statements. In practice, this means that researchers specify a simple sequence of controls resembling those of a tape deck—*Stop*, *Pause*, *Skip Forward*, *Skip Backward*, and *Shuffle*—together with three special controls: for *Surveys*, for sending *Emails*, and for *External* calls (e.g., for sending text messages). Not unlike tape decks, GOTO statements are fairly ancient technology, but we think they work well for the simple programming that most studies will require.

**Fig. 2:**
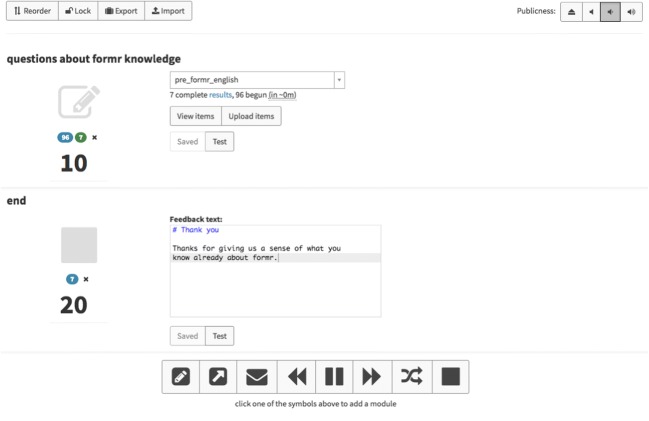
Screenshot of a very simple run for a one-shot survey. The tape deck controls at the bottom allow researchers to add further components, and the publicness settings for the study are in the top right-hand corner. The function of each button is explained on mouseover

All runs need to end in a Stop unit, which functions as the endpoint of the study. Stop units can be configured to show text to the user and can be used to give psychological feedback or tell users they cannot participate. In the latter case, there will be two stop buttons, one for those who cannot and one for those who can participate. A simple one-shot survey, such as those most commonly used in marketing research or cross-sectional psychological studies, would be a Survey unit followed by a Stop unit (see Fig. 2).

As an example of the more complex behavior that formr is capable of, consider a simple daily diary study, illustrated in Table 3.

**Table 3:** Schematic table of a diary study

Position Component	Description	Code/Text
10 Survey	Screening survey asking for demographics and contact information	Questions:age, gender, email address
20 SkipForward	Test whether participant is allowed to drink alcohol in the US, depending on their reported age.	screening$age >= 21 # If eligible for study, skip to position 40, if not go on to position 30.
30 Stop	If participant is younger than 21, stop them here.	Sorry, you are too young to participate.
40 Pause	Wait until tomorrow at 5 p.m. to start the diary.	You can close your browser now. You will be invited to continue by email tomorrow at 5 p.m.
50 Email	Invitation email to the diary	Please go to {{login_link}}
60 Survey	Daily diary assessing mood and drinking behavior	Questions: mood, drinking
70 SkipBackward	Repeat diary 30 times	nrow (diary) < 30 # if filled out fewer than 30 times, jump back to position 40
80 StopButton	End of diary	Thank you for participating. Here is how your mood and alcohol use correlate.```{r}library (ggplot2)qplot (diary$alcohol, diary$mood)```

The text after # shows comments in the R source code

For the study specified in this framework, almost anything can be customized. In the settings, researchers can change the link for the study, its title, its header image, and a footer for each page (e.g., providing contact information for the study team). Files can also be uploaded in order to include images, sound, or video elements in the study.

For more advanced researchers, custom CSS can be used to modify how the study appears to participants, and custom JavaScript can be used to modify dynamic interactions, and even to add browser-based reaction time tasks using jsPsych (de Leeuw, 2015) or lab.js, games, and so on.

At the level of the study framework, several logs are kept. One is the log of a user’s positions in the run, allowing researchers to track who was where and when. The software also logs sent emails and the results from automatic processing, such as API calls.

### Testing a study in formr

Because formr allows for fairly high complexity, we found it important to furnish researchers with tools to test that their studies operate as intended. Chief among them are test accounts. Study administrators can create new test accounts at the click of a button. They then receive a link that they can use themselves, as well as send to research assistants and co-researchers. The link defaults to a random animal name (this can be customized), so that administrators can test the study in different conditions or under different circumstances: For example, one could assign a student assistant the moniker *tenderUnicorn* to test the study as a single father, *awkwardTurtle* to test it as a teenager, *lazyStarfish* to contribute a lot of missing data in the diary, *slowCheetah* to respond very late after the diary invitation, *mobileMoose* to test on an Android smartphone, and so on. Testers can then report whether the study worked well under their testing conditions, whether any questions sounded odd to their persona, and whether the generated feedback made sense. Because entering data manually is often an unnecessary chore, especially for repeated surveys, test accounts automatically have a small “monkey bar” at the bottom of the survey. At the click of a button, testers can fill out the surveys using dummy data. They can also jump to different positions in the study sequence, delete their data, and end pauses prematurely. The data from these testers can be downloaded and checked for completeness.

Study administrators can also test surveys in isolation, but since this leaves out much of the complexity that tends to cause problems, we only recommend this for initial testing; researchers should thoroughly test complex studies before they are rolled out to real participants. Initially enrolling only a small number of participants can also be a wise strategy, for further, real-life testing. In longitudinal studies, we recommend keeping at least one tester enrolled for the duration of the study.

When the testing mode is turned on, any R code that is executed is tracked for debugging. If code errors occur, the code and error messages are automatically shown. If no code errors occur, testers can show the code using the magnifying glass and download it onto their computer to debug in a program such as RStudio (which has advanced debugging capabilities). If the testing mode is turned off, less informative error messages are shown, so as to avoid confusing real participants or accidentally disclosing too much information about the study.

### Monitoring an ongoing study

Longitudinal studies often require that communication with participants and special cases be managed. The main command center for this is the *Users* section in the study administration.^17^ The *Users* section offers a way to move users around in the run—for instance, to deactivate users or correct misplacements resulting from incorrectly configured control flows. It can also be used to send preformulated reminders. Perhaps most usefully, if participants report problems, the administrator can enter the study pretending to be the user (spy button) after enabling the testing mechanisms (stethoscope button) in order to gather more useful debugging information. Importantly, users are listed by their anonymous tokens, not by identifying information. If personal data have been properly separated from the research data as outlined above, it is possible to administer participants and fix problems without seeing personal data.

For some researchers, it can also make sense to program a custom R Markdown script in the *Overview* section, where they can define data aggregations and subsets to examine the data as they trickle in. Researchers can use formr to regenerate this report on the fly. It can be an additional tool with which to monitor data quality or even how much evidence has been accumulated for an a priori hypothesis, with a sequential-testing framework (Lakens, 2014; Schönbrodt, Wagenmakers, Zehetleitner, & Perugini, 2017).

### Sharing a study or run

**Fig. 3:**
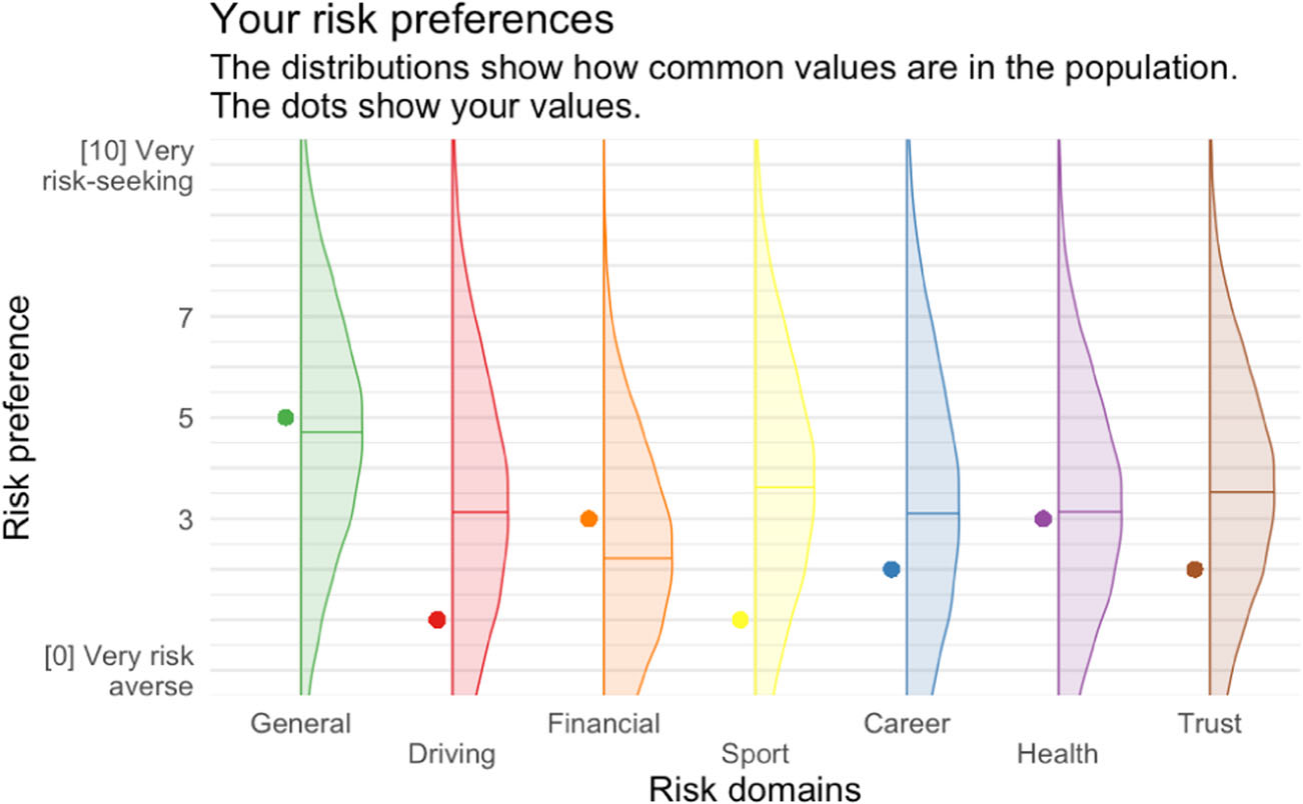
Feedback graph example from a study on risk preferences, showing a graph generated with ggplot2

Users can export the entire study with all settings, optionally including all survey items, as a JSON file. This makes it easy to share study designs with other researchers and allow them to reproduce and extend the work. Researchers can also design components such as a peer rating, a customized reminder, or personality feedback (see Fig. 3 for an example), so that other researchers can easily mix and match components to create new studies.

## R packages

Two R packages enhance the use of formr for researchers. The formr package provides some simple utility functions and idioms to users. It is automatically loaded whenever OpenCPU is called in formr. Outside formr, it can be used to easily load data from formr into R and to link the data to meta- and paradata.

On the basis of data pulled into R with the formr package (other sources are also possible), the codebook package (Arslan in press) generates metadata and data summaries that can be used as codebooks. This functionality helps formr users document their projects more easily and transparently. It pulls together information on the items and the results in order to generate graphs of item distributions and compute reliabilities of scale aggregates and general information on the survey datasets. In addition to being able to generate HTML, PDF, or spreadsheet-format codebooks, the data.frame objects in R are also marked up using this information, so that it is at the analyst’s fingertips in RStudio or when exported to SPSS and Stata. Metadata are additionally stored in JSON-LD format in the generated HTML documents, so that the dataset documentation is also machine-readable and discoverable for search engines.

## Case studies

To demonstrate the use of formr, we chose three case studies that the authors told us would have been difficult or impossible to realize using other open tools, short of programming customized studies or foregoing automation. All three led to articles that were published in the *Journal of Personality and Social Psychology*.

### A dyadic study with feedback for both partners

The formr software can be used to implement dyadic studies, such as studies involving both partners of a couple or best-friend dyads. In a recent multistudy investigation, Wurst et al. (2017) examined the effects of narcissism in early and later phases of romantic relationships. In particular, in their Sample O, they investigated actor and partner effects of narcissism using data from 272 committed couples. To collect these data, two separate runs—one for the first partner and one for the second partner—were implemented. Participants were recruited via email lists, online social networks, snowball sampling, and advertisements in lectures. Participants entered the first run and filled out a number of questions about their personality and their relationship. At the end of the surveys in the first run, participants were asked to share a personalized link with their partner in order to recruit them into the second run (the contents of the second run were identical to those of the first). This personalized link transmitted the partner’s email address, and optionally their personality data, although it would now also be possible to transmit such information invisibly using the experimental formr API.^18^ In addition, participants had to indicate whether they would be comfortable with their personality data being used for the dyadic feedback. After their partners had completed the second run, if they had also agreed to their data being used for the dyadic feedback, both partners received a dyadic personality profile.

### A longitudinal study with a social network component and other ratings

In the Göttingen Mate Choice Study (GMCS)—a multiwave investigation into partner preferences, relationship transitions, and relationship development—Gerlach, Arslan, Schultze, Reinhard, and Penke (2019) followed up on a large sample of singles. Using longitudinal data from the first two waves of the study, they investigated whether singles’ preferences predicted the characteristics of later partners. As such, the study’s prospective design was unique in the study of partner preferences. However, along with following up with participants longitudinally, the GMCS also incorporated further innovative features, such as a social network component and the assessment of participants and their partners through their peers.

In Wave 1, participants were screened to include only singles on the lookout for a potential partner. These participants initially answered some questions about their demographics, personality, and ideal partner preferences. They were then asked to provide a list of all potential romantic partners and to rate each on several attributes. Five months later, they received an email inviting them to the second wave of the study. In Wave 2, the authors reiterated the questions about ideal partner preferences and followed up on participants’ relationship experiences since Wave 1. These experiences included current and past partners, with both individuals from the network of potential partners and new people that participants might have met later. Crucially, by supplying the list of names from the previous wave as choice options in Wave 2, the authors could link the partners recruited from the network of potential partners with their Wave 1 ratings. In all cases, the authors obtained ratings on the same attributes for the chosen partners. Eleven months after the start of the study, the authors initiated Wave 3, which asked the individuals who had taken part in both previous waves to have their partners rate themselves and to have them rated by peers. This was implemented by setting up two additional studies (one for partners and one for peers), for which participants received links that included their participant code. Peers and partners who received this link via email were then invited to rate the partner and the original participant on the aforementioned attributes.

### A daily diary study with a social network component

The Daily Habits and Sexuality Study (Arslan, Jünger, Gerlach, Ostner, & Penke, 2016) collected data from 1,345 women over a period of 70 days. The women were first screened on a variety of demographic criteria. The main objective was to assess behavioral and psychological changes across the menstrual cycle. Thus, depending on whether the women were predicted to ovulate regularly according to a set of demographic and health criteria, they were offered either a monetary reward or entry in a lottery. Both rewards were calculated on the basis of an algorithm that took into account the frequency of participation and specifically rewarded a lack of large gaps in participation in the diary with a bonus. After being told which reward scheme they would receive, participants answered personality questionnaires and closed their browser.

Over the following 70 days, participants received a daily email invitation that told them how many days they had completed, how much money they had earned, and whether they had skipped the last day. If they had given their mobile phone number, they also received a text message reminder if they had not reacted to the previous day’s email or were in danger of losing their bonus.

In the diary, participants answered some items daily, and other items were randomly shown only on a subset of days, to keep the time needed to fill out the diary under 5 min per day while maintaining construct breadth (Revelle et al., 2017) and reducing rote responses.

Each day, single women noted who they had interacted with or thought about on that day (using first names or nicknames). The names of the interaction partners were cataloged using R. For the ten most mentioned names that were noted at least three times, women were asked to indicate whether these names belonged to men who were not their relatives. If they did, women answered a number of questions related to their attraction toward these men. This *loop* in formr continued until at least four unrelated men or at least ten persons in total had been assessed (assuming that enough names were mentioned in the diary). Unlike a standard social network questionnaire, which tries to elicit the names of interaction partners by relying on a participant’s memory and ability to generate names, this method made it possible to focus on the people who actually were the most commonly reported interaction partners in the diary period.

**Fig. 4:**
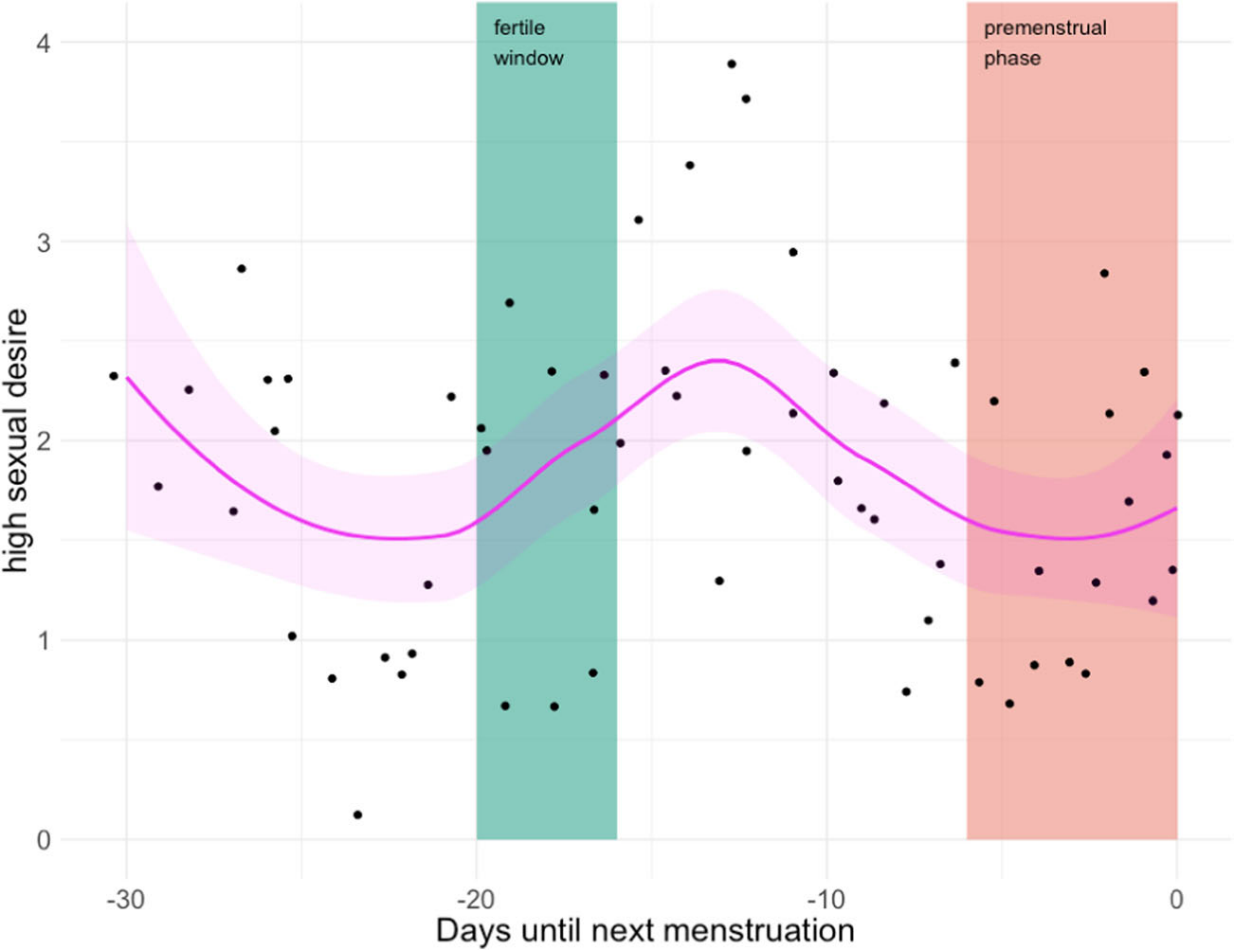
A (translated) graph of one participant’s psychological changes across the menstrual cycle in the daily diary study

**Fig. 5:**
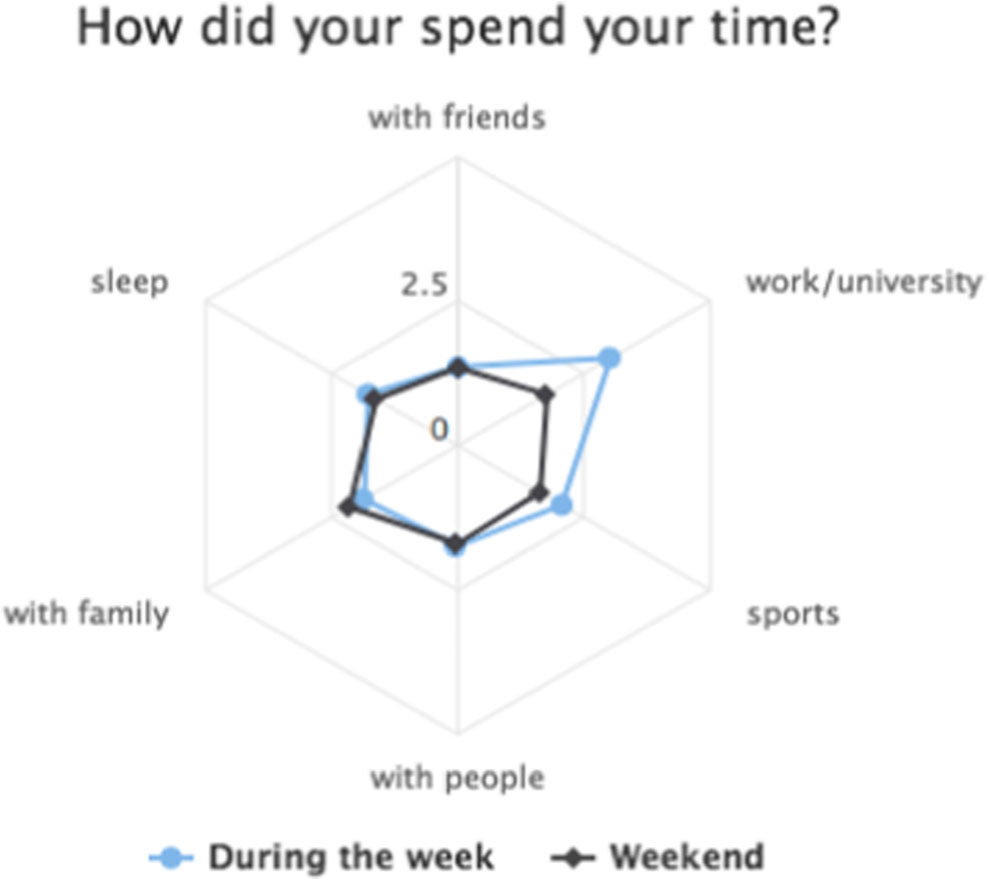
Example of a (translated) feedback graph, generated in the daily diary study to show how the participant had spent her time during the week and during the weekend

Participants then filled out a general follow-up questionnaire. In the end, they received automated, graphical, interactive feedback that included not only standard graphs, such as personality feedback, but also visual representations of how their mood, desire, and stress level varied across the menstrual cycle (see Fig. 4) and how they spent their time on different days (see Fig. 5). Scatterplots with fit lines were generated in order to let them examine whether there was any correlation between their alcohol consumption on the previous day and their sleep quality and mood the next day. They could also view the changes in their mood throughout the diary in an interactive display that allowed them to consult their notes on that day, to see why some days might have differed from others.

On average, women responded on 43 days during the diary period, and only 19% did not complete the follow-up (among those who were paid per response, the average response rate was 49 days, with 10% not completing the follow-up). Although the participants were not formally surveyed on this, online interactions indicated that the feedback was generally well received. The complete study is reproducible from documentation on the Open Science Framework and can be imported and changed (Arslan et al., 2016). An earlier, similar study implemented in an older version of formr was recently published and is described in more detail in Arslan, Schilling, Gerlach, and Penke (2018)**.**

### Other possible designs

The three case studies show how formr has been commonly used in ways that make full use of its feature set. However, we believe that a key strength of formr is that it can be used in ways that we did not plan for. For example, it would be possible to design an event-contingent monitoring study in which alerts are sent to users whenever a sentiment analysis shows an uptick in the use of emotion words on a social media platform. This would be possible because formr’s R support includes R packages that enable automated calls to the Twitter API and sentiment analysis. Similar applications could respond to weather events or events gleaned from screen-time monitoring apps. Contingent events could also be a partner’s or friend’s behavior in a dyadic design, evaluated using the formr API. A number of users have also used formr in the lab to obtain stimulus ratings and simple experiments, because they found randomization to be easy in formr. Some have even used formr as a vehicle for reaction-time-based experiments, by extending it using JavaScript.

## Running a formr server

It is possible to self-host an installation of formr on a university or commercial server. The installation instructions are detailed on Github. This allows researchers to more tightly control data privacy, lock the software version while a study is running, and customize domain and branding, but requires expertise to set up and maintain it securely. We may also recommend it to researchers who plan to run a very resource-intensive study, although this has not happened yet. The formr community supports researchers who plan to self-host formr.

## Community

The formr community of users has several forums for exchanging tips and information. The Github repository is the place to track issues such as bugs and feature requests. The formr wiki features a range of step-by-step how-to documents, contributed by users, that explain processes such as how to give simple feedback, implement peer ratings, embed videos, send text messages, or randomize items in blocks. The formr Google group is a mailing list through which users can search, ask, and answer questions. It is also where we announce upcoming changes and new software releases.

## Future directions

The formr software is still being actively developed. Since 2013, we have had about one major release every three months, and one minor release (bug fixes, etc.) every month. Despite the initial focus on surveys, we are now taking steps to make it easier to integrate formr with reaction time tasks, implemented in jsPsych or lab.js (Henninger, Shevchenko, Mertens, Kieslich, & Hilbig, 2019). A first study using jsPsych has been conducted, but we plan to offer more guidance on best practices in the future. We also want to gracefully handle users in different time zones. We also continuously monitor the performance of our servers and work on the scalability of formr by optimizing slow operations in our backend. For example, we plan a move to an architecture that will enable more fine-grained (at the second level) control over when messages are sent.

A more major planned feature is to integrate formr with openhumans.org (Price Ball & Greshake Tzovaras, n.d.) or midata.coop (E. Hafen, 2015), platforms that provide a way for people to share and link their research data from different sources (e.g., health trackers, phones, or commercial genetic-testing companies) while controlling anonymity. This would create a stronger firewall between research data and personal data than our existing methods do, while maintaining the ability to contact and remind participants and adding the capability to request data from other sources.

The formr software can already be connected to the Open Science Framework (OSF), thereby providing an easy way to back up the JSON file containing the study to the OSF and version-track it. We plan to make this integration tighter, to automate version tracking upon a change, and potentially even to allow researchers to preregister a design and track all subsequent ad-hoc changes, for greater transparency.

The biggest planned feature is to enable offline data collection and push notifications.^19^ Currently, we do not plan to support apps on multiple platforms, and core formr features such as the integration with R do not work offline. However, we hope to enable offline data collection by relying on progressive web apps (Progressive Web Apps, 2018), which are now available on all major mobile operating systems. This feature would also allow formr studies to function as apps, appear on the home screen, send notifications, and use system services such as the camera and the accelerometer. Although this would have to work without R, it would be useful for intensive experience-sampling research, for which an Internet connection should not be a prerequisite. Minor planned changes can be found on the formr Github page.^20^ We invite researchers who find formr’s feature set a useful starting point but not sufficient for their purposes to make sure any programmers they enlist via grant funding do work that can be openly shared with other users, too.

## Conclusion

With formr, we offer a simple way to collect data in longitudinal studies and give complex, immediate feedback. Its feedback and data analysis capabilities make formr useful not just for scientists, but also for therapists, Quantified Self enthusiasts, and others. Open-sourcing the software along with the study control framework and survey scripts helps increase the reusability, reproducibility, and transparency of research and makes it easier to collaborate.

### Author note

R.C.A. created the original software and wrote the first draft of this article. C.S.T. and M.P.W. improved and maintained the software and revised the manuscript. Numerous people and institutions have made formr possible, helped make it better, explored the boundaries of what it can do, and given support to the community. The authors thank Julia Zimmermann, Franz Neyer, the German Research Foundation (DFG), and the Thuringian Ministry for Education, Science and Culture for funding the initial project and allowing the code to be open source. We thank Lars Penke for funding work on formr, and Marc Reichardt for supporting its hosting. We thank all our users, especially our earliest and most committed users, who were patient when things went wrong and who suggested many useful features: Tanja Gerlach, Laura Botzet, Aileen Marske, Silvia Bradatsch, Julie Driebe, Stefanie Wurst, Sarah Lennartz, and Isabelle Habedank. We especially thank our power users Selina Reinhardt, Julia Rohrer, Julia Dauter, and Kai Horstmann for comments on an earlier version of the manuscript. We thank Deborah Ain for her thorough editing of the manuscript; all remaining errors are ours. Further information may be found at the following links: the hosted platform is at https://formr.org; the code is on Github, at https://github.com/rubenarslan/formr.org; and citable versions of the software are at https://zenodo.org/record/1345615.

## Footnotes

1 For example, Surveymonkey apparently allows no export of surveys, and Qualtrics encourages the use of a proprietary format. Neither supports the XLSform or Xforms standard, although Qualtrics does export nonstandard structured text files that are, with effort, machine-readable.

2 For example, Qualtrics apparently records location information, even when researchers use “anonymous links.” http://www.the100.ci/2018/07/25/student-research-projects-and-gdpr/.

3 For example, work on the QRTengine was discontinued after an update to Qualtrics broke the third-party extension and support was no longer responsive to its developers. (van Steenbergen, 2016)

4 For example, the vast majority of links to “free of charge” survey software on www.websm.org are either dead, link to software that has not been maintained for more than 2 years, or is no longer free.

5 With a lifespan of over 5 years and three active contributors, formr has exceeded the life expectancy and project health one would expect from examining the other free survey software listed on www.websm.org.

6 https://comparison-to.formr.org

7 https://www.opencpu.org/

8 https://en.wikipedia.org/wiki/Representational_state_transfer

9 Unfortunately, we know of no open-source alternatives to Google Sheets that provide a similar feature set. It is important to note that using Google Sheets to design surveys bears no risk of disclosing private participant information to Google unless the questions themselves contain such information. All formr does is to automate the download of an .xlsx file from Google; no data go in the opposite direction. If the questions contain sensitive information, a local spreadsheet editor such as LibreOffice or Microsoft Excel can be used.

10 https://widgets.formr.org

11 http://xlsform.org/

12 https://en.wikipedia.org/wiki/HTML

13 https://en.wikipedia.org/wiki/Markdown

14 https://R Markdown.rstudio.com/

15 https://www.w3.org/TR/html5/sec-forms.html#clientside-form-validation

16 https://en.wikipedia.org/wiki/Cascading_Style_Sheets

17 Many teams will also set up an email account for handling participant recruitment and contact. Since formr currently offers no way to exchange messages with participants, creating such an email account is a good idea for any larger study.

18 https://formr.org/documentation#api

19 https://en.wikipedia.org/wiki/Progressive_Web_Apps

20 https://github.com/rubenarslan/formr.org/issues
